# Supplementary material for: The Role of Surgery in Pleural Mesothelioma
Source: Cancers (Basel). 2024 Apr 28;16(9):1719. doi: 10.3390/cancers16091719 (PMC11083222; doi:10.3390/cancers16091719)
Supplement: Supplementary file 1 [file cancers-16-01719-s001.zip › cancers-2961131-supplementary.pdf]

Supplementary Table S1. Comparison between EPP and ePD.

	ePD		EPP
**Rate of tumor removal**		<	
**Perioperative morbidity rate**		<	
**Perioperative mortality rate**		<	
**Compliance with multimodality treatment**		>	
**Quality of life**		>	
**Long term survival**		≈	
**Locoregional recurrence rate**		>	

EPP-Extra pleural pneumonectomy, ePD-Extended Pleurectomy decortication.
